# Colcemid can double the ploidy of germ cells in female larvae, but not in male larvae, and only rarely in female adults.

**DOI:** 10.17912/micropub.biology.002126

**Published:** 2026-06-22

**Authors:** Lewis I. Held, Jr., Anushka V. Patil, Miguel Turrero García, Owen F. Vajnar, Cassandra G. O'pry, Kaitlyn N. Dewitt, Elman Behanfar, Alexis H. Shepard, Caroline G. Murphy, Aubriana M. Benson, Claire C. Carnevale, Pranav Chemudupaty, Karima H. Assal, Jamie F. Meuth, Kyle B. Baronia, Ria Umbrani, Lily M. Russell, Hariz A. Nawaz, Natasha N. Guhl, Hannah M. White, Jason J. Shin

**Affiliations:** 1 Dept. of Biological Sciences, Texas Tech, Lubbock, TX, United States

## Abstract

We successfully repeated an experiment where the anti-mitotic drug colcemid was fed to *Drosophila* larvae. Our aims were to (1) expand sample sizes, (2) improve survival at higher doses, (3) extend the treatment to adults, and (4) devise an improved method for assessing ploidy. We found that (1) colcemid has virtually no effect on germ cell ploidy in male larvae or female adults, (2) survival of larvae is increased by removing colcemid to allow metamorphosis, and (3) the wing margin offers a simple way to measure cell size, which is proportional to ploidy.

**Figure 1. Effects of feeding colcemid to larvae:** **a** . Inverse correlation (semi-log plot) of the fertility of colcemid-fed females (y axis) with the percent of adult triploid offspring (x axis). The trend line, which has a non-zero slope (p<0.0001), was plotted by linear regression (see Methods): y = 1.705*x + 164.7 (R ^2^ = 0.3861). **b** . Our new technique for measuring average cell diameter along the wing margin (mTR = medial Triple Row) in order to assess a fly's level of ploidy (see Methods). **c** . Differences in cell size along the wing margin of diploid (2n) vs. triploid (3n) flies. Blue triangles denote means. The 2n flies were females from a control *Dr/Sb* stock (64 wings total; mean = 15.8; s.d. = 0.68), while the 3n flies were female offspring from colcemid-fed adults (47 wings total; mean = 18.6; s.d. = 0.70). The 2n vs. 3n distributions are significantly different (p<0.0001; t test).

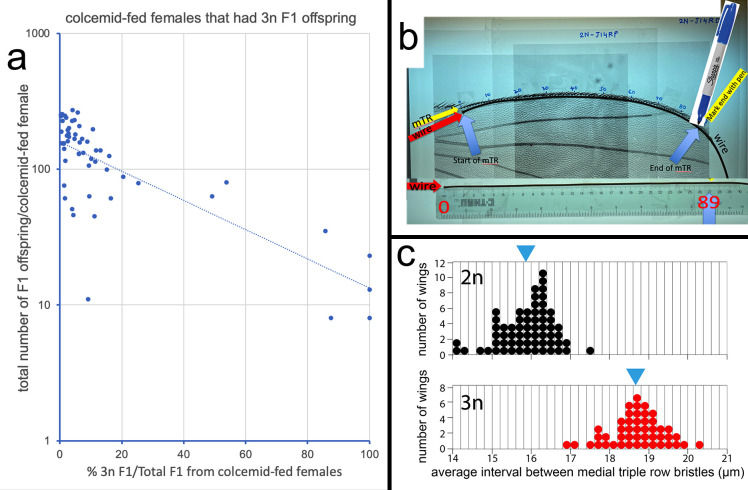



## Description

Polyploid flies offer a chance to probe how cell size affects anatomy because cell volume is proportional to ploidy in animals, and many structures (e.g., neural circuits) depend upon cell number, which is reduced in polyploids (Held, 2025). One of the simplest ways to increase ploidy in flies is to feed them colcemid, which disables the mitotic spindle. Colcemid’s ability to double the ploidy of germ cells in larvae was analyzed 44 years ago by one of us (Held, 1982), but the yield of polyploids fell sharply at higher doses. The percentage of triploid (3n) offspring peaked at 5 µg/ml (18%), but 90% of the mothers exposed to this dose died before adulthood. One of our goals in the present investigation was to devise a less toxic protocol.

Adults should be less sensitive than larvae to a drug like colcemid because they already have enough cells, whereas larvae must make more cells to build their bodies, and trying to do so while eating colcemid is as futile as Sisyphus trying to push a boulder uphill. Indeed, fly larvae delay metamorphosis until their imaginal discs grow large enough to complete that process (Texada et al., 2020). In contrast, *adult* females have been shown to survive 5 µg/ml of colcemid in their food for at least four days (Traut and Scheid, 1974), albeit with reduced fertility, so we decided to try treating adults instead of larvae.

1. Feeding colcemid to adults

Our colcemid-fed females had dominant markers ( *Dr* or *Sb* ) on their 3rd chromosomes and were mated with unmarked (wild-type) males, which allowed us to detect 3n F _1_ offspring by the presence of both traits (thin eyes and short bristles) in the same fly (see Methods). To our surprise, virtually no larvae were detected in the 1st 5-day brood at concentrations above 4 µg/ml, so we used drug-free food for the 2nd brood to try to restore fertility. This strategy worked, so we put the parents back onto colcemid for the 3 ^rd^ brood for one more exposure before the final drug-free broods (4 ^th^ & 5 ^th^ ). Table 1 presents the results.

Table 1. Number of F _1_ offspring obtained from colcemid-fed adult mothers*

**Table:** 

Colcemid dose (µg/ml)	Brood #1 (+ colcemid)	Brood #2 (no colcemid)	Brood #3 (+ colcemid)	Brood #4 (no colcemid)	Brood #5 (no colcemid)
0	7141 (0)	9035 (1)	5434 (1)	4396 (0)	5545 (0)
2	2723 (0)	8055 (1)	4802 (1)	5159 (1)	3247 (1)
4	306 (0)	5392 (0)	574 (0)	1149 (0)	193 (0)
6	21 (0)	8962 (3)	722 (0)	5423 (1)	2437 (1)
8	0 (0)	5941 (0)	97 (0)	3000 (1)	2482 (1)
10	0 (0)	5994 (6)	8 (0)	1979 (0)	1742 (11)

*Each cell in the table is the pooled total from the 5 vials in that cohort, each of which contained 50 mothers. In parentheses are the number of *Dr/Sb* (3n) offspring within the total (to the left). Broods lasted for 5 days, except for controls, whose (more fertile) parents had to be transferred every 4 days to avoid drowning in the scrum of larvae. The low survival of F _1_ in Broods #1 and #3 (where parents ate colcemid food) was not due to lower rates of egg laying by the parents, which appeared normal. Rather, the eggs failed to hatch (due to blocked mitosis?). Not tallied here were 7 +/+ (non- *Dr* , non- *Sb* ) offspring: 3 from controls, 1 from 2 µg/ml, and 3 from 8 µg/ml. These F _1_ flies likely arose from double crossover events between the *Sb* -bearing TM3 balancer and the *Dr* homolog—a conjecture supported by the fact that 10 +/+ flies occurred among 26,900 F _1_ from colcemid-fed female larvae, while only a single +/+ fly was found among 27,229 F _1_ from colcemid-fed male larvae. (Male flies in this species do not undergo *meiotic* recombination, though crossovers might rarely occur during germ cell *mitotic* divisions.)

A total of 30 *Dr/Sb* F _1_ flies was found: 28 from colcemid-fed mothers and 2 from controls. Four of the 30 *Dr/Sb* flies had to be 3n because they were intersexual (with sex combs, deformed genitalia, and incised wings), and the 3n status of the 26 *Dr/Sb* females was confirmed by measuring cell size along the wing margin (see Methods and Fig. 1c). A “burst” of 11 3n flies (9 females and 2 intersexes) was found in one of the five Brood #5 vials from the 10 µg/ml series, but other than this rare spurt, the distribution of 3n flies across other broods and doses was sporadic. The six 3n flies in Brood #2 of the 10 µg/ml series were distributed among 3 different vials (totals = 1, 2, and 3/vial).

2. Feeding colcemid to larvae

This weak effect of colcemid on adult females is basically a negative result, and negative results are hard to interpret. One remote possibility was that the drug itself was defective upon arrival from the manufacturer. To rule out this explanation, we decided to test a stock solution that was left over from the experiment to see if it was effective when fed to *larvae* (as an internal control). We tested the 6 µg/ml dose because it was tolerated by Brood #3 F _1_ larvae better than the higher (8 or 10 µg/ml) concentrations. We decided to further enhance survival by removing larvae from colcemid before pupariation.

Twenty vials of *Dr/Sb* females (50/vial) were crossed with Oregon R (wild-type) males (20/vial) and allowed to lay eggs for 5 days, with all of the hatched larvae allowed to feed on the colcemid-containing food until the 6 ^th^ day, at which point they were rinsed and transferred to fresh, drug-free food for the rest of their development. Five control (non-drug) vials were handled similarly. The 5 control vials yielded a total of 11,222 F _1_ offspring (mean = 2244 adults/vial), while the 20 colcemid vials only produced 468 F _1_ offspring (mean =23 adults/vial), which is two orders of magnitude fewer than the controls.

Each of the 179 surviving F _1_ colcemid-fed females was crossed with 2 Oregon R males, and their offspring were screened for *Dr/Sb* (3n) individuals. Ten of the 179 females (6%) were sterile (vs. 0% sterility among control females). The remaining 169 crosses yielded 26,890 offspring, of which 538 (2%) were both *Dr* and *Sb* . These 538 3n offspring (432 females and 106 intersexes) were distributed among 55 (33%) of the 169 mothers, with the number of 3n offspring/vial ranging from 1 to 43 flies/mother. The fertility of these 55 mothers was negatively correlated with the percentage of their offspring that were 3n (Fig. 1a).

Among the 20 colcemid vials, 7 (35%) were prepared with the same 6 µg/ml stock solution that was used in the *adult* -feeding experiment, and these crosses yielded 22/55 (40%) of the triploid-producing mothers and 239/538 (44%) of the 3n offspring, vs. the five 3n offspring from *adults* fed 6 µg/ml (Table 1). Based on this data, the low yield of 3n flies from adult (vs. larval) feeding can’t be attributed to defective colcemid. Possible reasons for the discrepancy in adult vs. larval sensitivity (aside from colcemid impotence) include: (1) adult females might digest colcemid in their gut before it can affect their germ cells, or (2) adult females might use a different (colcemid-resistent?) tubulin isoform in their spindles.

An equally baffling result was that no 3n F _1_ flies were obtained from colcemid-fed males. Among the 468 flies that survived a 6-day diet of 6 µg/ml colcemid during the larval period, 289 were males, and each of those was mated with 2 Oregon R virgins. Sterility was much greater than that of the females, with 62/289 (21%) of them failing to yield viable progeny. Among the remaining 227 fertile males, no *Dr/Sb* flies were recovered among their total of 27,229 offspring (nor were any found among 2,631 control F _1_ ). Why should the germ cells of these males be more resistant than those of their sisters, who were raised alongside them in the same vials? The disparity might be due to a male-specific (colcemid-resistant?) beta-tubulin isoform that exists in testes (Kemphues et al., 1982).

Alternatively, might diploid sperm be induced by colcemid but afterwards unable to fertilize eggs for some reason? In theory, yes, but this explanation is unlikely because compound-2, compound-3 fly stocks are known to be stable (Ashburner, 1989), presumably because nullo-2, nullo-3 eggs are regularly fertilized by 2n (double-compound) sperm.

## Methods

1. Feeding colcemid to adults

Five mg of colcemid was dissolved in 1 ml of 100% Ethanol and diluted in water containing Tegosept (fungicide). Six ml of the desired colcemid concentration was added to 3 mg of dry food flakes per vial with a calibrated syringe. The jelled food was compacted with a latex glove to ensure coherence during transfers. Grains of live yeast were sprinkled atop the food surface and wetted with the same colcemid dose before adding parental flies. Five concentrations of colcemid were used (2, 4, 6, 8, 10 µg/ml) plus the control (0 µg/ml). For each dose, 5 vials of parents were used: 50 virgin females and 10 wild-type males/vial. The strategy of detecting 3n F1 offspring by using the eye-shape mutation *Drop* ( *Dr* ) on one 3rd chromosome and the bristle-shape mutation *Stubble* ( *Sb* ) on the other (TM3 balancer) is 100% reliable (Held et al., 2025). The males were from an Oregon R stock. *Dr/Sb* virgin females were pre-treated with colcemid-containing food for 6 days before transferring them to fresh food with the same concentration and adding wild-type males. Further transfers to fresh (colcemid) food were performed at 5-day intervals, and the eggs from each brood were allowed to develop until eclosion, whereupon F _1_ adults were then inspected for 3n individuals based upon the presence of both *Dr* and *Sb* in the same fly.

2. Feeding colcemid to larvae

A single concentration of colcemid was used (6 µg/ml) for the entire experimental series, consisting of 20 food vials, each containing 50 virgin *Dr/Sb* females and 30 Oregon R males. Five vials of control flies (no colcemid in the food) were also established. Parents were allowed to lay eggs for 5 days, and all of the hatched larvae were rinsed and transferred to fresh, non-drug vials on the 6th day, where they were allowed to recover, re-commence mitosis, pupariate, and eclose.

3. Devising a better way to gauge cell size

The traditional way of assessing cell size is to count wing hairs (each secreted by one cell) in a sample square area in the middle of the fly wing (Dobzhansky, 1929), but this approach is hampered by (1) the fickleness of hair scattering from fly to fly and (2) the difficulty of distinguishing upper vs. lower surfaces of the wing in the plane of focus. We devised a more accurate method (Fig. 1b) by using the medial triple row (mTR) of the wing margin, where adjacent bristles are not separated by any intervening epidermal cells (Hartenstein and Posakony, 1989). We used a flexible wire to trace the curve of the mTR and then stretched the wire along a ruler to measure mTR length. Dividing that length (adjusted for magnification) by the number of bristle intervals in the mTR gives the average cell diameter (or a fixed multiple thereof).

We tested the accuracy of this approach by modeling cells as spheres. Given the proportionality of cell volume to ploidy (Fankauser, 1945), we expect the volume of a 3n cell to be 1.5 times that of a 2n cell. Hence, the *diameter* of a 3n cell should be the *cube root* of 1.5 (1.145) times the diameter of a 2n cell. If we multiply the mean interval between mTR bristles on 2n wings (15.8 µm) by 1.145, we calculate the *expected* interval between mTR bristles on 3n wings to be 18.1 µm. This number agrees well with the *observed* interval of 18.6 µm: the discrepancy (0.5 µm) is within one standard deviation (0.7 µm).

4. Statistics

To define the negative correlation between the fertility of colcemid-fed females and the proportion of 3n individuals among their offspring (Fig. 1a), we calculated the linear regression between these two parameters to obtain its equation. To compare the difference in cell diameter along the wing margin between 2n and 3n flies (Fig. 1c), we performed a two-tailed unpaired t test (p<0.0001), after verifying the normality of both datasets with the Shapiro-Wilk test (no statistical deviation was detected: p=0.053 for 2n; p=0.801 for 3n).

## Reagents

Colcemid was obtained from MP Biomedicals. Fly food (Formula 4-24) was purchased from Carolina Biological Supply.
